# The scent of offspring: chemical profiles of larvae change during development and affect parental behavior in a burying beetle

**DOI:** 10.1093/beheco/arae061

**Published:** 2024-08-09

**Authors:** Jacqueline Sahm, Beatrice Brobeil, Eric Grubmüller, Taina Conrad, Matthias Schott, Johannes Stökl, Sandra Steiger

**Affiliations:** Department of Evolutionary Animal Ecology, University of Bayreuth, Universitätsstraße 30, 95447 Bayreuth, Germany; Department of Evolutionary Animal Ecology, University of Bayreuth, Universitätsstraße 30, 95447 Bayreuth, Germany; Department of Evolutionary Animal Ecology, University of Bayreuth, Universitätsstraße 30, 95447 Bayreuth, Germany; Department of Evolutionary Animal Ecology, University of Bayreuth, Universitätsstraße 30, 95447 Bayreuth, Germany; Department of Animal Ecology I, University of Bayreuth, Universitätsstraße 30, 95447 Bayreuth, Germany; Department of Evolutionary Animal Ecology, University of Bayreuth, Universitätsstraße 30, 95447 Bayreuth, Germany; Department of Evolutionary Animal Ecology, University of Bayreuth, Universitätsstraße 30, 95447 Bayreuth, Germany

**Keywords:** begging, cuticular hydrocarbons, larval instars, *Nicrophorus*, parental care, volatile organic compounds

## Abstract

Chemical cues and signals, especially in insects, play a pivotal role in mediating interactions between individuals. Past studies have largely focused on adult semiochemicals and have neglected those of juvenile stages. Especially in the context of parental care, the larval odor might have a profound impact on parenting behavior, guiding parents in how much resources they should allocate to the different developmental stages. However, whether ontogenetic changes occur in subsocial species and whether larval-emitted scents influence parent–offspring interactions is largely unknown. Using 3 different sampling techniques, we analyzed the cuticular and VOC profile of the 3 larval instars of the burying beetle *Nicrophorus vespilloides*, which is known for its elaborate parental care. We found distinct differences in the cuticular and VOC profiles across the 3 larval stages. Second-instar larvae, which receive more frequent feedings from parents than the other larval stages, released greater amounts of acetophenone, methyl geranate, and octanoic acid isopropyl ester than the first and third instar. Additionally, using a newly developed bioassay with automated video tracking, we found that adding the odor of second-instar larvae to first-instar larvae increased the number of maternal feeding trips. Our results suggest that the odor produced by larvae plays an important role in mediating parent–offspring interactions. Given these findings, burying beetles might emerge as a promising candidate for identifying a potential begging pheromone.

## Teaser

Chemical substances released by insect larvae might be of high importance in parent–offspring interactions; however, the scents of juvenile stages have largely been neglected in research. We show that the chemical profiles of burying beetle larvae change during their development. Notably, second-instar larvae, which receive more attention from parents, emit higher amounts of certain compounds. The scent of these second-instar larvae increases parental feeding visits, indicating a crucial role for larval odors in parent–offspring dynamics.

## Introduction

Parental care occurs in many taxa and can be provided pre- or postnatally. Forms of care can range from protecting offspring from predators to providing food for the young (Clutton-Brock 1991; Balshine 2012; Smiseth et al. 2012; Trumbo 2012). Parental care increases offspring fitness (Trivers 1972; Smiseth et al. 2012; Wong et al. 2013) but also includes costs for parents in the form of used resources, time, and energy, and ultimately reduces their residual reproductive value (Alonso‐Alvarez and Velando 2012). To maximize the net benefit of care, recognition and communication between family members is essential (Ingold 1973; Hepper 1986; Waldman 1987, 1988; Clutton-Brock 1991; Bradbury and Vehrencamp 2011; Royle et al. 2012; Schultner et al. 2017; Steiger and Stökl 2017). For example, the ability to recognize offspring helps caring individuals to allocate resources toward their own instead of heterospecific or unrelated conspecific offspring (Kaplan et al. 1978; Cheney and Seyfarth 1980; Mateo 2002; Neff and Sherman 2003; Richard and Hunt 2013). While numerous species rely on location or temporal cues for offspring recognition, previous studies have demonstrated that many species dealing with brood parasitism possess the ability to distinguish between their own and heterospecific offspring using direct cues (Briskie et al. 1992; Lotem et al. 1992; Lyon 2003; Suzuki and Nagano 2006; Wang et al. 2008; Smith and Belk 2018). Furthermore, recognizing the nutritional state, age, or the developmental stage of offspring could be of critical benefit to both caregivers and offspring, as these attributes can determine the need for parental protection or food provisioning (Le Conte et al. 1994; Smiseth and Moore 2007; Smiseth et al. 2007; Traynor et al. 2015; He et al. 2016; Schultner et al. 2017). Parents might extract information about condition, age, or development stage by assessing the size of offspring or using other visual, chemical, or acoustic cues (Kilner 1995; Kölliker et al. 2005; Lévy and Keller 2009; Pelletier et al. 2016). However, in a range of parenting species, offspring are known to actively produce begging signals that reflect offspring need or quality and that influence the amount or duration of care (Kilner and Johnstone 1997; Royle et al. 2002; Kölliker et al. 2005; Mas et al. 2009; Mock et al. 2011). Given that the degree to which offspring rely on parental care depends on their developmental stage, the intensity or frequency of such begging signals typically changes as offspring grow (Davies 1976; Hirose and Balsam 1995; Smiseth et al. 2003; Jaeggi et al. 2008).

When it comes to insects, chemical cues and signals are the most widespread means of mediating interactions between individuals. Interestingly, although chemically mediated interactions have been intensively studied in several insect orders, these studies usually focused on adults and have largely ignored juvenile stages (Symonds and Elgar 2008; Wyatt 2008; Steiger and Stökl 2014; Oi et al. 2015; Yew and Chung 2015; Leonhardt et al. 2016; Pasqual et al. 2021; Buchinger and Li 2023). Currently, there are just a few studies available that examined chemical substances released by juveniles and even fewer that investigated whether there are qualitative or quantitative differences between development stages. For example, in a forensically important blowfly species, recent research has found differences in the composition of cuticular hydrocarbon (CHC) profiles (Sharma and Drijfhout et al. 2021) as well as the emission of volatile organic compounds (VOCs) (Sharma and Tomberlin et al. 2021) between different larval instars. A study of honey bee (*Apis mellifera*) broods also found a temporal change in the VOC profile during development (Noël et al. 2023). In this context, it has already been established that honey bee workers can discern the age of larvae based on VOCs, as evidenced by their different responses to the odors of young versus old larvae (Le Conte et al. 1994; Maisonnasse et al. 2010; Traynor et al. 2015). Contrary to our extensive knowledge about chemically mediated recognition and communication in eusocial insects (Leonhardt et al. 2016; Schultner et al. 2017; Schultner and Pulliainen 2020), our understanding of these processes in subsocial species that provide post-hatching care remains limited (Steiger and Stökl 2017; Nehring and Steiger 2018). Also, here, studies have typically focused on adults (Steiger 2015; Steiger and Stökl 2017), and to the best of our knowledge, there is no study that analyzed how chemical profiles change during development. There are, however, studies suggesting the existence of chemical begging signals. Research has shown that food deprivation has an effect on the quantity of specific cuticular hydrocarbons in earwig nymphs (Mas et al. 2009) and on VOC profiles in burrower bugs (Kölliker et al. 2006). In order to deepen our understanding of chemical substances released during offspring growth and to establish a foundation for future investigations into the chemistry of parent-offspring interactions, we conducted an in-depth study on the chemical profiles of different larval stages using the burying beetle *Nicrophorus vespilloides*, known for its elaborate pre- and post-hatching care, as a model system. To this end, we analyzed larval cuticular lipid profiles using solvent extraction and VOC profiles using active and passive headspace techniques. In addition, we established a suitable bioassay to show that larval odor affects parental behavior.

Burying beetles provide elaborate biparental care for their offspring using small vertebrate carcasses as a breeding resource (Pukowski 1933; Eggert and Müller 1997; Scott 1998; Royle et al. 2013). Monopolized carcasses are transformed into a ball-like shape, whilst removing fur or feathers, and treating the carcass with anti-microbial secretions to prevent decomposition (Suzuki 2001; Cotter et al. 2010; Arce et al. 2013; Vogel et al. 2017; Shukla et al. 2018; Miller et al. 2019). Additionally, beetles cut a hole into the prepared carcass, in which larvae aggregate either to be provisioned by their parents or feed themselves (Eggert and Müller 1997; Eggert et al. 1998; Scott 1998; Smiseth et al. 2003; Royle et al. 2013; Trumbo 2017). Food provisioning appears to be primarily triggered by larval tactile begging (Rauter and Moore 1999; Smiseth et al. 2003). Hereby, larval begging increases with hunger (Smiseth and Moore 2007) and proximity to parents (Smiseth and Moore 2004). *Nicrophorus* larvae pass through 3 instars during development (Pukowski 1933), with each instar exhibiting differences in size, begging rate, and dependency on parental food provisioning (Eggert et al. 1998; Smiseth et al. 2003). In particular, it is known that second-instar larvae show the highest tactile begging rate and are the most frequently fed among the 3 instars (Smiseth et al. 2003). However, it is currently unknown whether larvae produce chemical cues or signals that reflect their developmental stage and, therefore, their dependency on parental food provisioning.

Previous studies showed that chemical cues and signals play an important role in *Nicrophorus* beetles. For instance, they use VOCs emitted by decaying carcasses to locate their breeding resources over long distances (Kalinová et al. 2009; Trumbo and Steiger 2020). Furthermore, VOCs and cuticular lipids are used by adults to identify sex, previous mating partners, and their breeding partners (Steiger et al. 2007, 2009; Steiger et al. 2008; Haberer et al. 2010, 2014; Chemnitz et al. 2015; Keppner et al. 2017) and therefore play an important role in social interactions. Moreover, brood-caring females produce a volatile (methyl geranate) that reflects their hormonal state and acts as an anti-aphrodisiac to males (Engel et al. 2016, 2019). It has also been suggested that breeding beetles emit chemical stimuli that triggers begging behavior in larvae (Smiseth et al. 2010; Takata et al. 2019). In the case of the offspring, there are certain hints that parental beetles differentiate between the development stages of larvae. For example, Takata et al. (2013) showed that brood regulation mostly concerns newly hatched larvae reaching the carcass. Furthermore, Engel et al. (2016) showed that when females were regularly provided with first-instar larvae, females continued to care for their given offspring instead of producing future offspring. But when faced with third-instar larvae, females resumed egg laying (Engel et al. 2016). Additionally, studies have also found corresponding effects of the larval stage on maternal juvenile hormone titers (Scott and Panaitof 2004; Trumbo and Robinson 2008). There are also some indications that parents base care decisions on chemical cues produced by larvae. Mattey et al. (2018) showed that females provide different amounts of care for inbred and outbred larvae. They hypothesize that inbred larvae produce a signal based on which females recognize their poor genetic condition and suggest that this signal is of chemical nature (Mattey et al. 2018). Furthermore, various studies found that parents can evaluate brood size. They resume egg laying when brood size is very low (Sahm et al. 2022) or cull some offspring when there are more larvae than the resource can support (Bartlett 1987; Trumbo and Fernandez 1995; Smith et al. 2015). It is possible that parents use the amount of VOCs released by larvae for such decisions. Moreover, it is also known that parents of some *Nicrophorus* species are able to discriminate between own and heterospecific larvae, a behavior likely mediated by chemical cues (Capodeanu-Nägler et al. 2018; Smith and Belk 2018). Hence, investigating the production of VOCs and cuticular lipids by *Nicrophorus* larvae, and determining whether they reflect their developmental stages, could be of key importance to better understand parent-offspring interactions during family life.

To examine the chemical profile of *N. vespilloides* larvae, we collected VOCs and cuticular lipids from all 3 instars. We predict that the chemical profiles of the first and second instar differ from that of the third instar, given that the first two stages are much more dependent on parental provisioning than the latter. Furthermore, if larvae express a chemical begging signal, we predict that second-instar larvae differ in their chemistry as they are fed more frequently by the parents and also show a higher tactile begging behavior compared to the other 2 instars (Eggert et al. 1998; Smiseth et al. 2003). In the passive headspace samples, we detected methyl geranate (MG), but it was unclear if larvae themselves produce MG or whether it is a residual from females who are known to produce MG in the presence of larvae. To verify that larvae actively produce MG, we exploited the fact that females only produce MG when caring for offspring with a male partner (Steiger et al. 2011; Engel et al. 2016). Hence, we additionally analyzed the MG emission of larvae raised either uni- (single females) or biparentally (female and male). Finally, we tested whether the parental feeding rate is affected by the odor of larvae.

## Material and methods

### Beetle origin and husbandry

We used larvae of *Nicrophorus vespilloides* beetles that originated from outbred populations kept in our laboratory at the University of Bayreuth, Germany. Beetles descended from wild-caught beetles captured near Bayreuth, Germany and were kept in small plastic boxes (10 × 10 × 6 cm) filled with moist peat and stored in a climate chamber at 20 °C under a 16:8 h light: dark cycle. Beetles were fed with sliced mealworms (*Tenebrio molitor*) twice a week.

### Solvent extractions

To produce larvae for the solvent extraction (and the active headspace), we randomly paired unrelated, virgin males and females in plastic boxes (10 × 10 × 6 cm) half-filled with moist peat. Pairs were given access to a weighted mouse carcass of approximately 20 g. After 48 h, we separated the eggs form their parents, by transferring beetles and carcasses into a new, similar-sized box filled with moist peat. Twenty-four hours later, we checked the boxes containing the eggs for hatching larvae every hour. We analyzed the chemistry of all 3 different larval instars. Larvae of the first first instar were either 0 h (newly hatched) or 6 h old, larvae of the second instar were 24 h old, and larvae of the third instar were either 48 or 72 h old (larval mass, mean ± SD, L1: 3.28 mg ± 1.02; L2: 17.22 g ± 3.25; L3: 132.8 g ± 58.95). Therefore, newly hatched larvae were either directly subjected to chemical analysis, or larvae were assigned to their own parents for 6, 24, 48, and 72 h before they were analyzed.

To extract the cuticular lipids from the surface of the larvae, we adjusted our approach based on size and weight differences among the instars. We pooled 5 larvae from the first instar, 3 larvae from the second instar, and used a single larva for each of the third instars, placing them in a 1.5-ml glass vial for the extraction. Before conducting solvent extractions, we collected the active headspace of the larvae (see below). The larvae were then freeze-killed at −20 °C. Following this, we added *n*-hexane (Rotisolv, Carl Roth, Karlsruhe, Germany) as a solvent to dissolve the cuticular substances on their surface. Due to size differences of larval instars, we added 300 µl *n*-hexane to the first- and second-instar larvae for 5 min and we added 1000 µl *n*-hexane to the third-instar larvae for 3 min. Afterwards, the extracts were transferred into new, 1.5 ml glass vials, and the larvae were discarded. We evaporated the extracts under a gentle nitrogen stream to a volume of approximately 50 µl. Then we added 1 µl *n*-hexane containing 20 ng Eicosane (Sigma-Aldrich, St. Louis, USA) as internal standard and auto-injected 1 µl of each extract splitless into the GC-MS (Shimadzu GC2030 gas-chromatograph connected to a Shimadzu QP2020NX mass-spectrometer; Shimadzu, Duisburg, Germany). The GC contained a non-polar capillary column (SH-Rxi-5Sil MS, length = 30 m, inner diameter = 0.25 mm, film thickness = 0.25 µm, Shimadzu, Duisburg, Germany), and the oven temperature was raised from 40 °C to 300 °C at a rate of 5 °C/min and finally held for 20 min. We used helium as a carrier gas (linear velocity = 50 cm/s). *n*-alkanes were identified through a comparison of their mass spectra and retention indices with a reference mixture of alkanes (Sigma-Aldrich, St. Louis, USA). Other CHCs were identified by interpretation of the MS spectrum and comparison of the retention index with the literature (Carlson et al. 1998). Other compounds were identified by comparing their mass spectra and linear retention indices with the NIST database. We characterized the positions of the double-bonds in mono- and diunsaturated compounds by analyzing samples derivatized with dimethyl disulfide samples (Carlson et al. 1989) in the GC-MS system as described above. Additionally, we compared the retention indices of the unsaturated substances of the larvae with those identified previously in adults (Steiger et al. 2007).

### Active headspace analysis

Generally, headspace describes the gas phase, for example, around an object, in our case, around the larvae. For the active headspace, we pumped the gas phase actively into our collective medium, whereas for the passive headspace (see below) we collected the headspace without using an active force, but by placing a fiber above the larvae to collect the VOCs via diffusion.

For the collection of the active headspace of larval instars, we used the same larvae as for the solvent extractions. Each sample was placed in a silanized glass jar (inner diameter = 3 cm) containing a wet filter paper to prevent larval desiccation. The glass jar was connected to a “headspace filter” and a membrane pump as well as an activated charcoal filter to clean incoming air (50 mg; Supelco, PA, USA). Headspace filters consisted of a 2-cm long glass tube (inner diameter = 2 mm) enclosed on both ends with silanized glass wool (Sigma-Aldrich, Supelco, St. Louis, USA) and contained 3 mg of Carbotrap® B and 3 mg Tenax® (both Sigma-Aldrich, Supelco, St. Louis, USA). Before usage, filters were conditioned using a Clean Cube (SIM, V1.0, Oberhausen, Germany). Headspace analyses were conducted in a climate chamber at 20 °C. Here, larvae were placed inside the glass jars for 20 min to accumulate their volatiles before we sucked air through the jar using the pump (~200 ml/min) to collect volatiles for 5 min. Afterwards, headspace filters were stored at −20°C in a freezer till further analysis. Prior to analysis, we added 20 ng of methyl undecanoate (Sigma-Aldrich, St. Louis, USA) dissolved in 1 µl of *n*-hexane (Rotisolv, Carl Roth, Karlsruhe, Germany) as an internal standard. Headspace filters were desorbed (300 °C for 8 min) using a thermal desorption system (TD-30R, Shimadzu, Duisburg, Germany) connected to a GC-MS system as described above. The oven temperature was raised from 50 °C to 200 °C at a rate of 5 °C/min before raised to 280 °C at a rate of 15 °C/min, which was then held for 10 min. Helium was used as carrier gas (linear velocity = 36.3 cm/sec). Prior to the comparison of the active headspace chemistry of larval instars, we removed siloxanes and other contaminations from our analyses. Given the absence of discernible variations in the chemistry between larval instars (see results), we opted against conducting further in-depth characterizations of the substances detected.

### Passive headspace analysis

To generate larvae for passive headspace sampling, beetle pairs were given access to a mouse carcass (~8–12 g), and we checked the boxes after 72 h for hatching larvae every 2 h. We collected either five first-instar larvae (newly hatched larvae), five second-instar larvae (24 h old), or five third-instar larvae (48 h old) in 4 ml glass vials.

Furthermore, we collected passive headspace samples from second-instar larvae raised under uni- or biparental care for 24 h to specifically investigate their MG production. We focused on second-instar larvae as they showed the highest amount of MG (see Results). Previous studies showed that females caring for second-instar larvae produced the highest amount of MG if their male partner was present (Steiger et al. 2011; Engel et al. 2016). Under uniparental care, females produce no or only trace amounts of MG. Hence, collecting larval headspace volatiles under uni- and biparental care allows us to investigate if MG is produced by the larvae or whether it is just transferred from biparental caring females to the larvae. We randomly set up 20 unrelated pairs of males and females of *N. vespilloides* beetles in plastic boxes (10 × 10 × 6 cm) half-filled with moist peat and provided them with a carcass of approximately 10 g. Forty-eight hours after beetles had access to the carcass, males were removed in half of the boxes to create uniparental caring females. About 24 h later, we checked the boxes for larval hatching every 2 h. After a parental care period of 24 h, we collected five second-instar larvae from each family and placed them separately in a 4-ml glass vial.

We collected the passive headspace of each sample described above using SPME (= Solid-Phase Microextraction). At first, we created an opening in the lid of the 4 ml glass vials containing the collected larvae of each instar/treatment using an injection needle. Here, we inserted a SPME fiber (PDMS/DVB, 65 µm, fused silica, 24Ga, Supelco, Bellefonte, USA) which was used to collect larval volatiles for 30 min. SPME fibers were desorbed in the injector of the GC, which was coupled to the MS, for 5 min at 250 °C. The GC contained a non-polar column (like the column used in “Solvent extractions”). Starting at 50 °C (held for 2 min) we raised the oven temperature of the GC to 280 °C with a rate of 5°C/min to separate the volatiles. Helium was used as carrier gas (linear velocity = 40 cm/s). Afterwards, SPME fibers were conditioned in the GC-MS injection port at 250 °C for 30 min before being used again. Further, a calibration curve was created by applying varying amounts of synthetic methyl geranate (5–200 ng/µl) to a filter paper inside a 4-ml glass vial and sampling the methyl geranate with SPME fibers for 15 min inside a fume hood. The identification of the substances was achieved by comparing their mass spectra and linear retention indices with those of synthetic reference compounds.

### Arena experiments

To test whether the parental feeding rate is affected by the odor of larvae, we established a behavioral choice assay that allowed us to measure the response of caring females using automated video tracking. To this end, we exploited the fact that females feed their larvae also outside of the carrion resource; specifically, they move from the cadaver to a different site and regurgitate food to them (J. K. Müller, personal communication). A validation experiment served to test the suitability of the choice assay and a subsequent experiment to test the response of females to the surface extracts of larvae.

For both experiments, we paired unrelated virgin males and females in plastic boxes (10 × 10 × 6 cm) half-filled with moist coconut coir and provided each pair with a mouse carcass (8.5–12.5 g). After 48 h, we separated the eggs from the parents by transferring the females and carcasses into a new, similarly sized box filled with moist peat. The males were removed at this time point as our aim was to focus on female behavior. Twenty-four hours later, we checked the boxes containing the eggs for hatching larvae every hour. Once the larvae had hatched, we transferred the corresponding mothers along with her carrion resource into an arena, which was designed to offer the females a binary choice. The arena consisted of a rectangular plastic box (12 × 12 × 6 cm) filled with a thin layer of moistened plaster with 3 round, shallow impressions of different sizes (Fig. S1). A larger one in the corner of the arena, in which a medium petri dish (94 mm diameter, 16 mm height) was placed, and two smaller ones in close proximity, matching 2 smaller petri dishes (35 mm diameter, 10 mm height). Both impressions containing the smaller petri dishes were at the same distance and angle from the larger petri dish. Females, along with their carrion resource, were consistently placed in the larger petri dish, while larvae were positioned in the smaller ones. To prevent the larvae from escaping, the inner walls of the smaller petri dishes were treated with Antlock (Antstore, Berlin). Additionally, a damp piece of paper towel was placed inside to maintain humidity for the larvae. Each arena was sealed with an anti-reflective glass pane that had been sprayed with antifog (Cressi, Barcelona, Spain). This design not only prevented the females from escaping but also facilitated video tracking. All arena experiments were conducted in a dark climate chamber under red light and at 20 °C.

To validate the suitability of the arena assay, we tested the females’ response to 1 versus 10 first-instar larvae. Larvae were randomly drawn from a pool of newly hatched larvae and transferred to the 2 petri dishes. We then counted the number of visits of the females at the 2 petri dishes for 8 h using video recordings and an automated analysis technique (see details below). As we expected, females spent more time at the petri dish with 10 larvae rather than 1 larva (see results); we therefore performed a subsequent experiment. We tested the females’ response to the surface extract of larvae compared to a control. To obtain the larval extract, we used second-instar larvae, as they are fed most frequently by the parents. The larvae had been reared biparentally for 24 h. For each septum, we extracted a batch of 20 larvae in 700 µl *n*-pentane (Rotisolv, Carl Roth, Karlsruhe, Germany) for 3 min. Each larval extract was evaporated to approximately 10 µl and applied to the septum. Because the second-instar larvae used for extraction were raised on a carrion resource and thus had been in contact with carrion substances, we prepared an extract of carrion odors as the control. For this, we rubbed both the in- and outside of a parentally prepared cadaver with 5 filter paper pieces (area of 1 cm^2^), extracted them using 5 ml *n*-pentane for 3 min and applied 10 µl to a silicone GC septum (Septa-N8, diameter = 1.3 mm, Macherey-Nagel, Dören, Germany). Using a silicone septum offers an advantage in that it provides a constant emission of substances over a longer time period compared to the use of filter paper, for instance (Engel et al. 2016). For the experiment, one of the petri dishes was then equipped with a silicone septum soaked with larval extract, while the other had a septum soaked with the control extract. Furthermore, we placed 2 first-instar larvae in each of the petri dishes to provide an additional stimulus for the females and to give them the opportunity to feed the larvae. Again, we counted the number of visits of the females at the 2 petri dishes for 8 h using video recordings and an automated analysis technique (see details below).

### Video analysis

For the video recordings, HD TVI mini cameras (BSC TVI 2811, 2.8–12 mm, Eutin, Germany) were used, with a frame rate of 25 and a resolution of 960 × 576 pixel. The cameras were connected to a recording device (LUPUS - LE918 4K 8 Channel NVR, LUPUS-Electronics GmbH, Landau, Germany). Analyses of the recorded videos utilized a custom-built Python script (Version 4.3). The Python script enables the user to manually select round regions of interest (ROIs) from the first frame of the video. The software then isolates these ROIs (here the 2 petri dishes; Fig. S1), frame by frame, and conducts a comparison between successive frames. In instances where no motion occurs, ROIs remain black; however, movement is represented by the conversion of the moving pixels to white. The script assesses motion by measuring the proportion of white pixels relative to the total ROI area, which gives a percentage of the area that was active over time.

The resulting patterns of activity in the video data, denoted by spikes, were indicative of movement. To differentiate between the movement of the females and the larvae, we established a specific activity threshold. Activities above this threshold were attributed to the beetles, while those below it were ascribed to the larvae. To determine these thresholds, we analyzed the plots alongside the actual behavior of the beetles as observed in the videos. This analysis indicated that a threshold of 65 was appropriate for the validation experiment, which involved comparing 10 larvae to one. For the subsequent experiment that included 2 larvae and additional chemical cues, a lower threshold of 30 proved to be sufficient (Fig. S2). To establish the number of female visits per petri dish, the peaks with a maximum above the respective threshold in all plots of a recording were determined. Note that the automatic video tracking technique cannot detect whether a female visit actually involves feeding or not.

### Statistics

All statistical analyses were performed in R (version 4.2.2, R Core Team). When analyzing the chemical profiles of the different larval instars, we always used the relative amounts of the substances. We removed all substances from the dataset which represented less than 0.5% of the total peak area before we standardized each profile to 100%. We identified 42 cuticular substances in the surface extracts (*N*_oh_ = 17, *N*_6h_ = 8, *N*_24h_ = 22, *N*_48h_ = 25, *N*_72h_ = 20; summary in Table S1) and 45 volatile substances in the active headspace samples (*N*_oh_ = 16, *N*_6h_ = 10, *N*_24h_ = 18, *N*_48h_ = 22, *N*_72h_ = 16). For our passive headspace samples of the 3 instars, we focused on the 10 most prominent volatile organic compounds (= VOCs) (*N*_oh_ = 18, *N*_24h_ = 16, *N*_48h_ = 20; summary in Table S2). For the VOCs, we additionally calculated the absolute amount of each substance per larva prior to the analysis.

To determine if larval instars can be separated based on cuticular lipids or VOCs deriving from active or passive headspace, we calculated 3 PERMANOVAs (= Permutational analysis of variance; “adonis2()” command in the “vegan” package) as well as pairwise PERMANOVAs (“pairwise.adonis()” command in the “pairwiseAdonis” package; Bonferroni-corrected p-values) based on Bray-Curtis-dissimilarities. Additionally, we visualized the data using nMDS (= non-Metrical Multidimensional Scaling) plots based on Bray-Curtis-dissimilarities in the R-package vegan and heatmaps (“heatmap.2()” command in the R-package gplots. Finally, we calculated SIMPER tests (‘simper()’ command) for each dataset. SIMPER tests show the contribution of each substance to the differentiation between larval instars. For the substances contributing most to the differentiation of larval instars, we further tested whether their amount differed significantly between larval instars using Kruskal–Wallis tests followed by pairwise Wilcoxon tests with Bonferroni correction. Lastly, we analyzed the difference in the amount of MG between uni- and biparental raised second-instar larvae using a Wilcoxon rank sum test (*N* = 10 each).

For the arena experiments, we compared the number of visits of females (a) between the petri dish containing 1 larva versus the petri dish containing 10 larvae (*N*_1vs10_ = 17) and (b) between the extracts of L2 larvae and the extracts of the carrion (*N*_L2vsCarrion_ = 12) using paired Wilcoxon tests.

## Results

### Cuticular lipids of the 3 larval instars

**Fig. 1.**
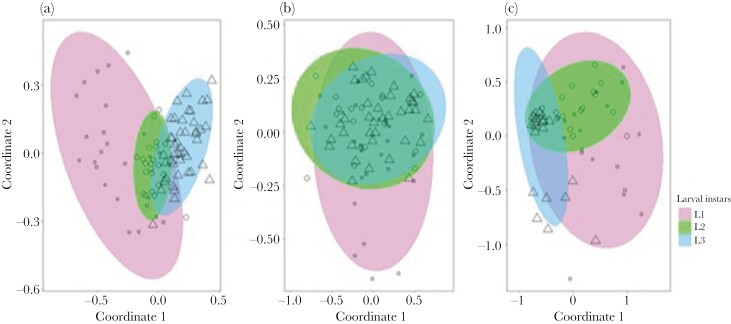
NMDS ordination based on Bray-Curtis dissimilarities of (a) the chemical profile of the solvent extractions from 3 larval instars (*N* = 92), (b) the chemical profile of the active headspace analysis from 3 larval instars (*N* = 82), and (c) the chemical profile of the passive headspace analysis from 3 larval instars of *N. vespilloides* (*N* = 54). Each symbol represents the chemical profile of one sample. Confidence ellipses denote 95% confidence areas around the group centroid.

**Fig. 2.**
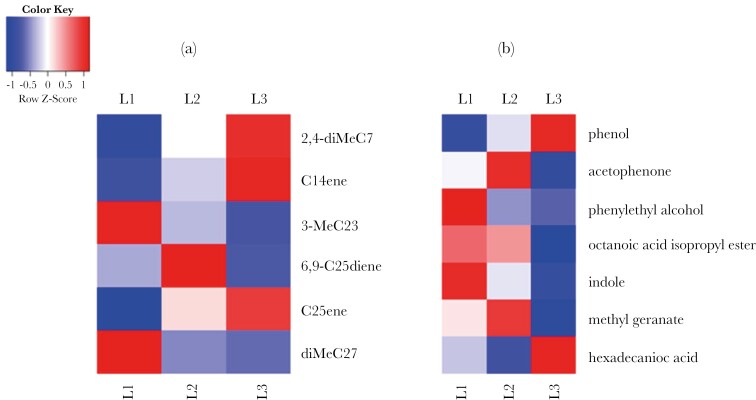
Heatmap of the relative amount of (a) the 6 substances found in the surface extractions that contributes most to the differentiation of larval instars based on Simper-tests (see results) and (b) the 7 substances of the passive headspace analysis showing significant differences between larval instars (see Results). The color of the squares reflects the row z-score and, therefore, the deviation from the mean quantity of each substance across all larval instars: red shades denote values above the mean (higher quantity), blue shades indicate values below the mean (lower quantity), and white represents values near the mean.

**Fig. 3.**
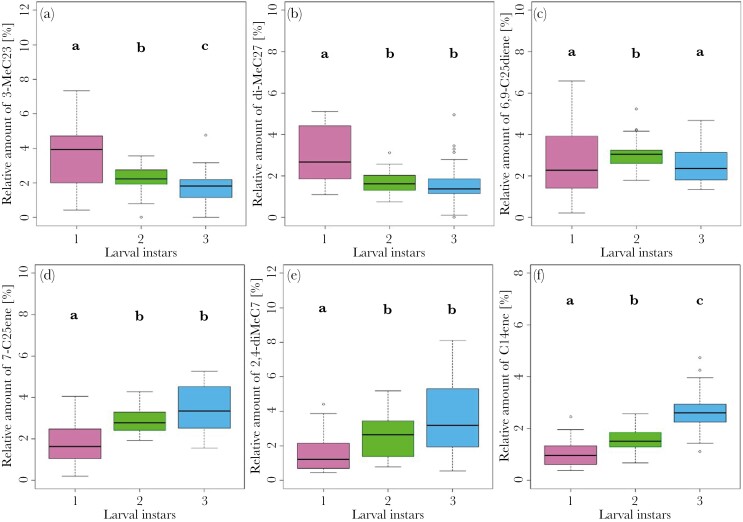
Boxplots showing the 6 surface substances which showed the highest contribution (based on Simper-tests) in the differentiation between larval instars. Shown are the relative amount of (a) 3-MeC23, (b) diMeC27, (c) 6,9-C25diene, (d) 7-C25ene, (e) 2,4-diMeC7, and (f) C14ene for the 3 larval instars of *N. vespilloides*. Different letters indicate significant differences after Bonferroni correction.

The solvent extractions revealed 42 substances of *N. vespilloides* larvae from different instars (Table S1). We found differences in the cuticular lipid profile between larval instars (PERMANOVA, *F* = 27.18, *P* = 0.001; Fig. 1a). Thereby all instars differed from each other (for each pairwise PERMANOVA, *F* > 11.9, *P* < 0.01). SIMPER tests showed that diMeC_27_ (SIMPER test; 0.71) contributed highly to the separation of the first-instar larvae from the second- and third-instar larvae (Fig. 2a). Furthermore, between the 1^st^ and second instar predominantly 6,9-C_25_diene (SIMPER test; 0.66) and 2,4-diMeC_7_ (SIMPER test; 0.69) separated the instars, whereas between the first- and the third-instar 3-MeC_23_ (SIMPER test; 0.67) and 7-C_25_ene (SIMPER test; 0.69) contributed most to the separation (Fig. 2a). The substances contributing most to the differentiation of the second and third instar were C_14_ene (SIMPER test; 0.66), 7-C_25_ene (SIMPER test; 0,69), and 6,9-C_25_diene (SIMPER test; 0.71; Fig. 2a). Analyzing the substances separately, we found that larval instars differed in their produced levels of 3-MeC_23_ (Kruskal–Wallis test, χ^2^ = 22.75, *P* < 0.001; Fig. 3a), diMeC_27_ (Kruskal–Wallis test, χ^2^ = 25.33, *P* < 0.001; Fig. 3b), 6,9-C_25_diene (Kruskal–Wallis test, χ^2^ = 6.33, *P* = 0.04; Fig. 3c), 7-C_25_ene (Kruskal–Wallis test, χ^2^ = 25.51, *P* < 0.001; Fig. 3d), 2,4-diMeC_7_ (Kruskal–Wallis test, χ^2^ = 21.74, *P* < 0.001; Fig. 3e), and C_14_ene (Kruskal–Wallis test, χ^2^ = 55.79, *P* < 0.001; Fig. 3f). Larvae of the first instar showed higher relative amounts of 3-MeC_23_ and diMeC_27_ compared to the other instars (pairwise Wilcoxon test, *P* < 0.01). For the second instar, we found higher amounts of 7-C_25_ene, 2,4-diMeC_7_, and C_14_ene compared to the first instar (pairwise Wilcoxon test, *P* < 0.02), and higher amounts of 3-MeC_23_ and 6,9-C_25_diene compared to the third instar (pairwise Wilcoxon test, *P* < 0.02). Lastly, third-instar larvae showed higher amounts of 7-C_25_ene, 2,4-diMeC_7_, and C_14_ene compared to the first instar (pairwise Wilcoxon test, *P* < 0.001) and higher relative amounts of C_14_ene compared to the second instar (pairwise Wilcoxon test, *P* < 0.001).

### VOCs of the 3 larval instars

In our active headspace samples of larvae, we found 45 substances. However, our analysis revealed no differences between larval instars based on their active headspace chemistry (PERMANOVA, *F* = 1.94, *P* = 0.07; Fig. 1b).

**Fig. 4.**
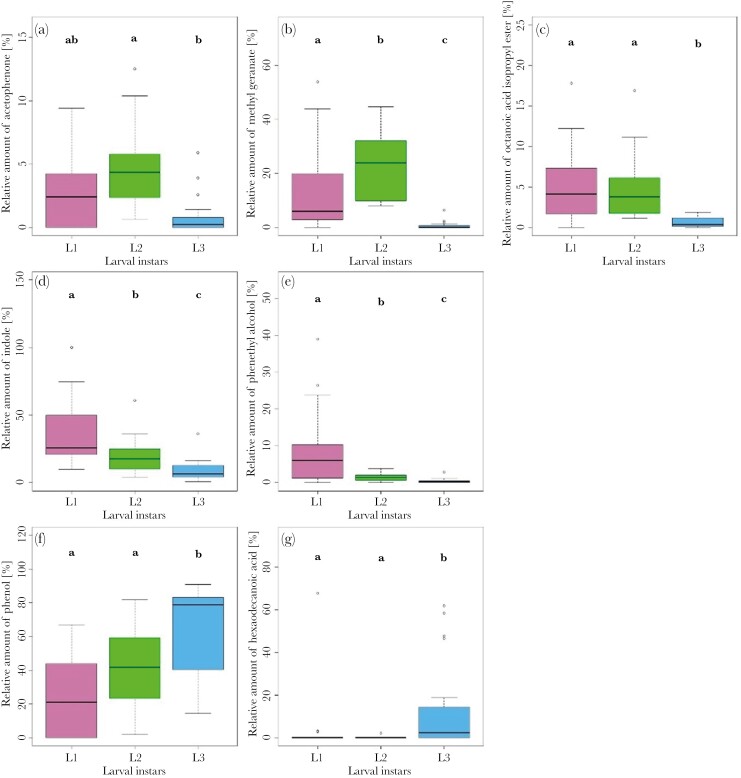
Boxplots showing the 7 passive headspace substances differing between larval instars. Shown are the relative amount of (a) acetophenone, (b) methyl geranate, (c) octanoic acid isopropyl ester, (d) indole, (e) phenethyl alcohol, (f) phenol, and (g) hexadecanoic acid for the 3 larval instars of *N. vespilloides*. Different letters indicate significant differences after Bonferroni correction.

For analyzing the passive headspace of larval instars, we focused on 10 substances (Table S2). Our analyses revealed an overall difference between larval instars based on their passive headspace chemistry (PERMANOVA; *F* = 12.83, *P* = 0.001; Fig. 1c). Pairwise comparisons showed that all 3 instars differed from each other in the relative amount of VOCs produced (pairwise PERMANOVA, *F* > 4.7, *P* < 0.02). SIMPER tests showed that the instars were predominantly separated based on methyl geranate (SIMPER test; > 0.5) and indole (SIMPER test; > 0.5; Fig. 2b). In addition, the first and second instars are separated by phenol (SIMPER test; 0.30) and the second and third instars by hexadecanoic acid (SIMPER test; > 0.65; Fig. 2b). Testing the substances separately, we found that larval instars differed in their produced levels of acetophenone (Kruskal–Wallis test, χ^2^ = 17.11, *P* < 0.001; Fig. 4a), methyl geranate (Kruskal–Wallis test, χ^2^ = 33.53, *P* < 0.001; Fig. 4b), octanoic acid isopropyl ester (Kruskal–Wallis test, χ^2^ = 23.02, *P* < 0.001; Fig. 4c), indole (Kruskal–Wallis test, χ^2^ = 25.22, *P* < 0.001; Fig. 4d), phenethyl alcohol (Kruskal–Wallis test, χ^2^ = 19.45, *P* < 0.001; Fig. 4e), phenol (Kruskal–Wallis test, χ^2^ = 19.00, *P* < 0.001; Fig. 4f), and hexadecanoic acid (Kruskal–Wallis test, χ^2^ = 20.71, *P* < 0.001; Fig. 4g). First- and second-instar larvae produced relative higher levels of methyl geranate, octanoic acid isopropyl ester, indole, and phenethyl alcohol compared to third-instar larvae (pairwise Wilcoxon test, *P* < 0.05). Furthermore, second instars produced more acetophenone than third instars (pairwise Wilcoxon test, *P* < 0.001), and third instars produced a higher amount of hexadecanoic acid and phenol than larvae from other instars (pairwise Wilcoxon test, *P* < 0.02). For the relative amounts of 2-ethyl-1-hexanol, ethyl caprylate, and quinoline, no differences were found between larval instars (pairwise Kruskal–Wallis test, *P* > 0.07). Interestingly, even when we calculated the absolute amount of substances emitted per larva, we found that the second-instar larvae produced a higher amount of acetophenone (pairwise Wilcoxon test, *P* = 0.004), methyl geranate (pairwise Wilcoxon test, *P* < 0.001) and octanoic acid isopropyl ester (pairwise Wilcoxon test, *P* < 0.001) than the larger third-instar larvae, and they also emitted a higher amount than the first-instar larvae.

**Fig. 5.**
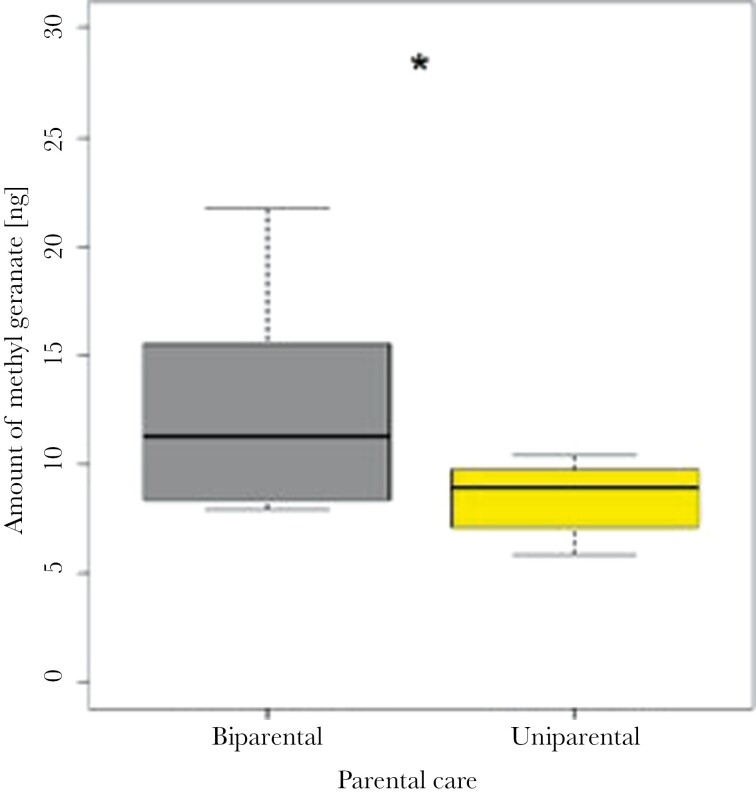
Boxplots showing the absolute amount of methyl geranate produced by larvae raised either under uni- or biparental care. Values represent the absolute amount of MG collected from 5 larvae. Asterisk indicates the level of significance (*P < 0.05).

We detected methyl geranate in the headspace of uniparentally raised second-instar larvae, indicating that the larvae produce methyl geranate themselves. However, the amount of methyl geranate measured was higher under biparental than uniparental care (Mann–Whitney *U* test, *W* = 77, *P* = 0.04; Fig. 5).

### Arena experiments

**Fig. 6.**
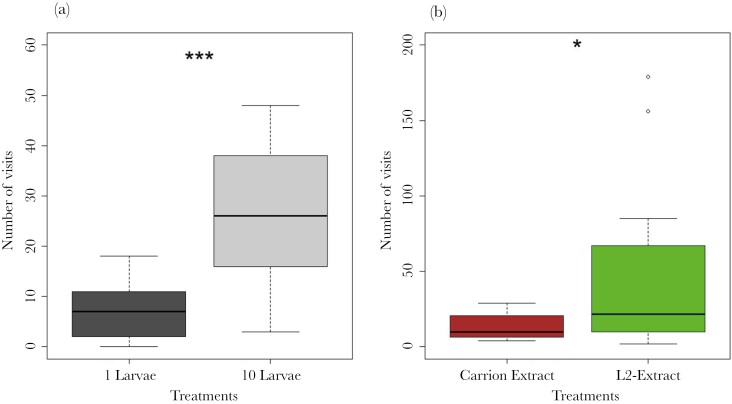
Boxplots showing the number of female visits over an 8-h period to petri dishes containing (a) 1 larva or 10 larvae (*N* = 17) and petri dishes containing (b) 2 larvae and either carrion or L2 extracts (*N* = 12). Asterisks indicate the level of significance (*P < 0.05; ***P < 0.001).

We found that females visited petri dishes with 10 larvae more often than petri dishes with 1 larva (paired Wilcoxon test, *V* = 6, *P* < 0.001; Fig. 6a). Females also preferred petri dishes containing extracts of L2 larvae to those containing carrion extracts (paired Wilcoxon test, *V* = 6, *P* = 0.01; Fig. 6b).

## Discussion

Our chemical analyses revealed that (1) burying beetle larvae produce a diverse set of cuticular lipids and VOCs, (2) both the composition of the cuticular profile and the VOC profile differs between the 3 larval instars, (3) larvae produce methyl geranate (MG), the same volatile as breeding mothers, and (4) second-instar larvae, which are known to be fed more frequently by the parents than the other 2 instars, emit higher amounts of acetophenone, MG and octanoic acid isopropyl ester than the first and third instar. We were also able to establish a suitable bioassay using automated video tracking to better understand the role of larval odor in parent–offspring interactions. We found that adding the odor of second-instar larvae to first-instar larvae increased the number of maternal feeding trips. Taken together, our results suggest that chemical cues (or even signals) produced by larvae play an important role in mediating parent–offspring interactions in burying beetles.

In the solvent extract of larvae, we detected 42 substances, mostly cuticular hydrocarbons. Although this suggests a less complex profile compared to adults, which exhibit over 90 substances (Steiger et al. 2007; Keppner et al. 2017), it is possible that lower quantities in larvae resulted in some substances being below our detection threshold. While we found no qualitative differences in the cuticular profile across the 3 instars, we did notice quantitative variations. The first-instar larvae had higher amounts of 3-MeC_23_ and diMeC_27_, while the second-instar larvae produced more 6,9-C_25_diene, and the third-instar larvae had a higher concentration of C_14_ene than the other 2 instars. Interestingly, changes in the CHC profile during development have also been observed in other necrophagous insects. For instance, in several forensically important species of the fly families Calliphoridae and Sarcophagidae, different larval development stages differ in their CHC composition (Zhu et al. 2006; Roux et al. 2008; Pechal et al. 2014; Sharma and Drijfhout et al. 2021; Zhang et al. 2022). Studies revealing age-dependent changes in CHC composition are, however, not limited to carrion insects; such changes have also been observed in Lepidoptera larvae, for instance (de Renoables and Blomquist 1983). Given that all these studied species lack parental care, the observed changes in CHCs might not have a communicative function in parent-offspring interactions, suggesting the influence of other factors. Sharma et al. (2021) and Zhu et al. (2006) found age-related shifts from shorter to longer chained CHCs in blowfly larvae and interpreted these shifts as a potential adaptation that prepares older larvae for survival in drier environments. This could also be true for burying beetle offspring, as third-instar larvae leave the carcass to pupate in the drier soil. However, our data do not show an ontogenetic shift from shorter to longer chained CHCs, suggesting that other factors may be influencing the CHC profile. Although we cannot currently rule out that the observed changes in CHC profiles are merely metabolic byproducts, it is possible that these changes serve a communicative function. Particularly when considering that cuticular lipids play a fundamental role in guiding interactions among burying beetle adults (Steiger et al. 2007, 2009; Steiger and Franz et al. 2008; Keppner et al. 2017), it seems reasonable that larvae might use them to communicate their developmental stage or age to their parents.

We did not only find differences in CHC compositions, but the VOC profile also differed between the 3 larval instars. The differences were only detected when analyzing the passive headspace samples but not the active ones, a finding that underlines the value of implementing diverse sampling methods to obtain a more detailed picture of VOCs produced by insects. A likely reason for the difference between the sample methods could be that the different absorbents used vary in their efficiency in absorbing larval volatiles. Like the cuticular profile, the VOC profile did not show any qualitative differences, only quantitative ones. Of particular interest was that the second instar larvae emitted higher amounts of acetophenone, methyl geranate, and octanoic acid isopropyl ester compared to the other 2 instars. Given that parental food provisioning typically peaks during the second instar (Smiseth et al. 2003), these VOCs could potentially act as begging pheromones, enhancing the effectiveness of the tactile begging behavior. That VOCs can vary with age and function as potential begging pheromones, mediating interactions between larvae and caregivers, was also shown in previous studies in honey bees (Traynor et al. 2015; He et al. 2016; Noël et al. 2023). Specifically, (E)-β-ocimene, emitted in higher quantities by younger larvae compared to older ones, influences worker foraging (Traynor et al. 2015), and its production increases when larvae are food-deprived (He et al. 2016).

We found that *N. vespilloides* larvae produce MG, the same substance as that produced by caring mothers. Maternal MG has been shown to play a key role in regulating mating and care behavior in burying beetles (Engel et al. 2016, 2019; Royle 2016). During the time of intensive brood care, when parents are tending to young larvae, females do not produce any further eggs (Engel et al. 2016; Sahm et al. 2022). During this time, they emit MG, which reliably reflects their reproductive state and functions as an anti-aphrodisiac inhibiting male mating behavior (Engel et al. 2016). Interestingly, it is the interaction with the young larvae that triggers maternal MG emission and prevents females from producing further eggs. This is evident from the fact that removing the brood or replacing it with older, third-instar larvae results in the cessation of MG emission and a resumption of egg laying as long as sufficient carrion resources are available. It is known from (E)-β-ocimene in honey bees that it does not only regulate worker provisioning behavior but also inhibits egg production (Maisonnasse et al. 2009). Hence, it is possible that larval MG also has such a dual function, acting as a begging signal and preventing mothers from allocating resources into egg production (Steiger and Stökl 2018). In fact, the concept of such begging pheromones with both releaser effects on behavior and primer effects on maternal reproductive physiology was anticipated earlier by Mas and Kölliker (2008), suggesting their prevalence in brood-caring insects. However, an alternative possibility is that larvae emit MG to enhance its anti-aphrodisiac effect, since it is in the larval interest that both parents care for them and are not distracted by matings. This hypothesis could also explain the observed higher MG emission from larvae raised in biparental conditions compared to those raised by females alone. Future studies are needed to unravel the function of larval MG.

It is certainly unlikely that all the VOCs we have identified are involved in parent-offspring interactions. One or several substances might mediate interactions between larvae, for example, serve as an aggregation pheromone. This could aid newly hatched larvae in locating the carrion resource more easily, fostering communal feeding for the brood’s benefit (Schrader et al. 2015; Prang et al. 2022). Larval aggregation pheromones have been found, for example, in flies (Mast et al. 2014), moths (Jumean Z et al. 2005; Díaz-Siefer et al. 2021), bugs (Chen and Liang 2015), or locusts (Torto et al. 1996; Wertheim et al. 2005). It is also likely that some of the substances found in the larval headspace have no communicative function. Substances like phenol, acetophenone, phenylethyl alcohol, indole, and hexadecenoic acid have also been detected in the secretions of adults and might be released by larvae due to their antimicrobial properties (Degenkolb et al. 2011; Haberer et al. 2014).

Finally, we were able to establish a suitable bioassay by exploiting the fact that females also feed their larvae outside the carrion resource. This off-nest feeding allowed us to implement a binary choice test coupled with automatic video tracking. We found that females visited 2 larvae supplemented with larval extract more frequently than those supplemented with the control extract. Thus, our bioassay demonstrates that females respond to larval-derived odors and supports our notion that interactions between parents and offspring are driven, in part, by chemical cues or signals. However, based on this current data, we cannot say which of the chemical components have a communicative function, nor what kind of information the mothers are extracting. It is possible that they simply use chemical compounds to estimate the number of larvae. If the parents prefer to feed larger broods and utilize these chemical cues to assess brood size, this could explain the observed differences in visiting rates in our bioassay. However, it is also possible that they use them to assess age, nutritional state or other qualitative aspects of larvae. Given these possibilities and considering our data alongside the theory on the evolution of begging signals, we believe that burying beetles represent promising candidates for identifying a potential begging pheromone that influences parental investment.

In conclusion, our results highlight the importance of studying the scent of juvenile stages, thereby considering both cuticular lipids as well as more volatile substances. The strong focus on the chemistry of adults in the last decades has hampered our understanding of the role of larval semiochemicals. Through our research, we have demonstrated that the composition of chemical profiles undergoes developmental shifts, suggesting that such ontogenetic changes are likely to be widespread across insects. Moreover, our study has successfully established the importance of larval-derived odors in mediating parent-offspring interactions in burying beetles. We hope that these findings will encourage future studies to test the factors that drive the age-related chemical plasticity, as well as to test the significance of single compounds or mixtures emitted by larvae. Offspring semiochemicals are likely to be heavily involved in the regulation of family life.

## Supplementary Material

arae061_suppl_Supplementary_Material

## Data Availability

Analyses reported in this article can be reproduced using the data provided by Sahm et al. (2024).
