# Supplementary material for: The scent of offspring: chemical profiles of larvae change during development and affect parental behavior in a burying beetle
Source: Behav Ecol. 2024 Aug 9;35(5):arae061. doi: 10.1093/beheco/arae061 (PMC11319877; doi:10.1093/beheco/arae061)
Supplement: arae061_suppl_Supplementary_Material [file arae061_suppl_supplementary_material.docx]

**Supplementary material**

**The scent of offspring: chemical profiles of larvae change during development and affect parental behavior in a burying beetle**

Jacqueline Sahm, Beatrice Brobeil, Eric Grubmüller, Taina Conrad, Matthias Schott, Johannes Stökl, Sandra Steiger


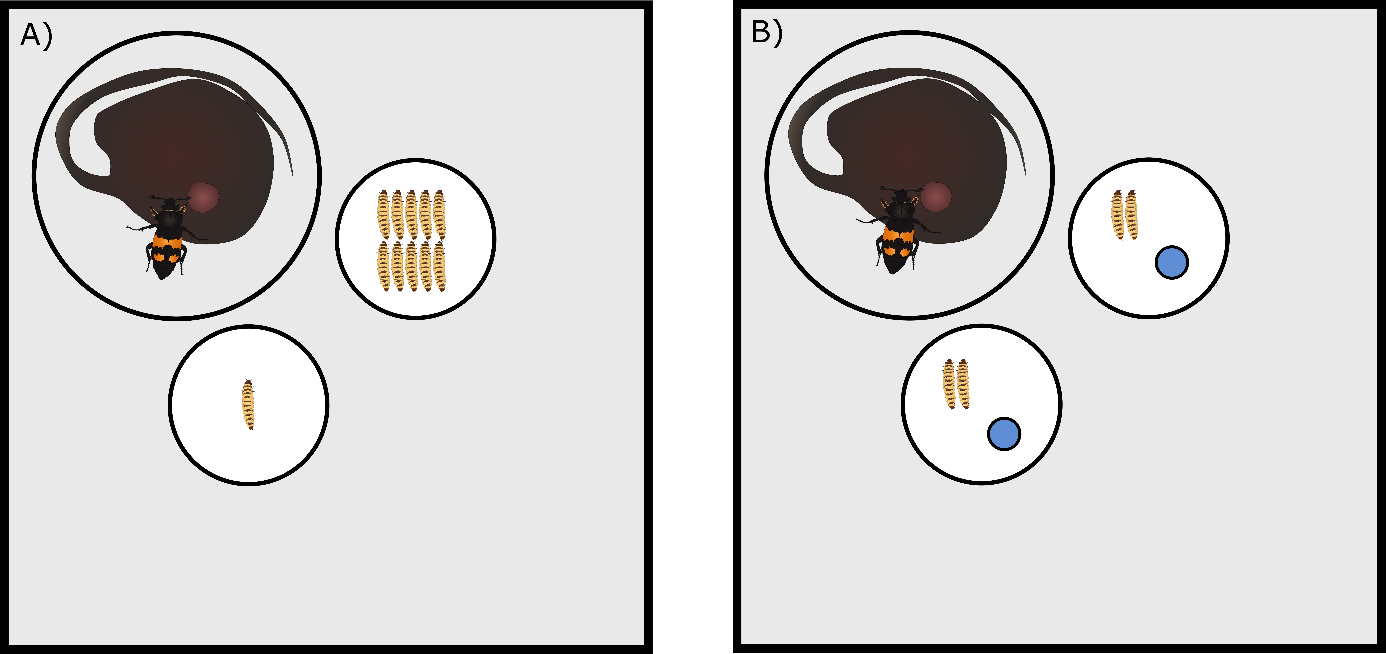


**Figure S1**: Illustration of the arena design. A) shows the set up to validate our arena experiment by placing either 1 larva or 10 larvae in a small petri dish for a female to choose from. In B) we added 2 larvae per petri dish and a septum containing either the carrion extract as a control or the extract of larvae from the 2nd instar.


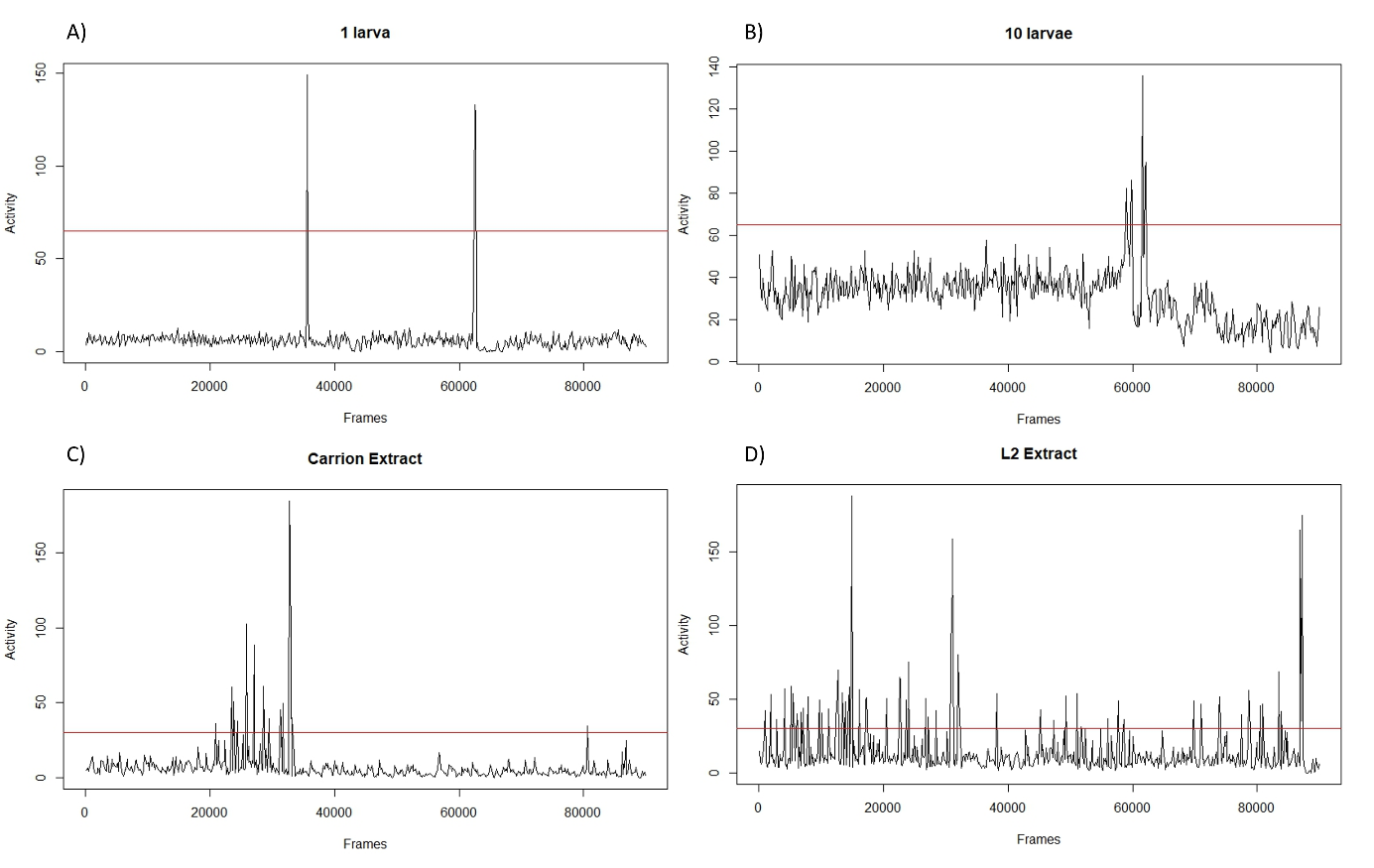


**Figure S2**: Plots show the patterns of activity in form of spikes which indicate the movement of females. A) and B) represent the activity patterns of the validation trials where females had the choice between 1 larva and 10 larvae, respectively. C) and D) show the activity pattern of females choosing between carrion extracts and L2 extracts, respectively. Red lines represent the thresholds defined for the activity of females. In A) and B) an activity value over 65 and in C) and D) an activity higher than 30 were defined as a response from the female to the treatments.

| **Table S1:** Mean and standard deviations of the relative contribution (%) of specific substances found in the surface extracts of *N. vespilloides* larvae from three different larval instars separated by GC (mean relative peak area). Mass spectra of unidentified substances (marked with a *) are provided at the end of the supplement. | | | | | | |  |
| --- | --- | --- | --- | --- | --- | --- | --- |
| Peak number | Retention time | Retention index | Substance | 1st instar | 2nd instar | 3rd instar |  |
| 1 | 4.90 | 813 | 2,3,5-triMeC_6_ | 0.21 ± 0.17 | 0.38 ± 0.22 | 0.51 ± 0.32 |  |
| 2 | 5.07 | 819 | 2,4-diMeC_7_ | 1.61 ± 1.11 | 2.64 ± 1.31 | 3.75 ± 2.05 |  |
| 3 | 6.09 | 861 | 4-MeC_8_ | 0.41 ± 0.28 | 0.66 ± 0.29 | 0.96 ± 0.44 |  |
| 4 | 11.79 | 1057 | 4-MeC_10_ | 0.82 ± 0.53 | 1.32 ± 0.50 | 1.99 ± 0.79 |  |
| 5 | 11.96 | 1061 | 3-MeC_10_ | 0.15 ± 0.15 | 0.28 ± 0.22 | 0.42 ± 0.17 |  |
| 6 | 13.10 | 1102 | unknown MeCHC* | 0.37 ± 0.26 | 0.55 ± 0.25 | 0.89 ± 0.35 |  |
| 7 | 13.27 | 1108 | unknown MeCHC* | 0.11 ± 0.12 | 0.18 ± 0.12 | 0.36 ± 0.16 |  |
| 8 | 15.56 | 1190 | C_12_ene | 0.60 ± 0.37 | 0.97 ± 0.38 | 1.49 ± 0.54 |  |
| 9 | 17.37 | 1273 | nonanoic acid | 0.15 ± 0.29 | 0.94 ± 0.73 | 0.97 ± 0.62 |  |
| 10 | 18.23 | 1279 | 3-MeC_12_ | 0.44 ± 0.35 | 0.67 ± 0.28 | 1.14 ± 0.38 |  |
| 11 | 18.84 | 1295 | Indole | 0.19 ± 0.23 | 0.43 ± 0.21 | 0.60 ± 0.28 |  |
| 12 | 19.07 | 1300 | *n*-C_13_ | 0.13 ± 0.21 | 0.33 ± 0.19 | 0.51 ± 0.26 |  |
| 13 | 19.30 | 1323 | unknown MeCHC* | 0.12 ± 0.23 | 0.33 ± 0.21 | 0.52 ± 0.26 |  |
| 14 | 19.43 | 1326 | unknown MeCHC* | 0.25 ± 0.23 | 0.44 ± 0.17 | 0.65 ± 0.22 |  |
| 15 | 20.99 | 1390 | C_14_ene | 1.05 ± 0.53 | 1.57 ± 0.44 | 2.64 ± 0.72 |  |
| 16 | 23.78 | 1500 | *n*-C_15_ | 0.09 ± 0.14 | 0.10 ± 0.13 | 0.38 ± 0.14 |  |
| 17 | | 23.45 | 1512 | dimethyl butylphenol | 2.11 ± 1.21 | 3.10 ± 0.82 | 5.56 ± 1.36 |
| 18 | 24.84 | 1549 | MeC_15_ | 0.09 ± 0.17 | 0.20 ± 0.22 | 0.45 ± 0.36 |  |
| 19 | 25.05 | 1564 | Unknown* | 0.12 ± 0.20 | 0.23 ± 0.22 | 0.42 ± 0.23 |  |
| 20 | 25.86 | 1592 | C_16_ene | 0.89 ± 0.48 | 1.30 ± 0.32 | 2.22 ± 0.55 |  |
| 21 | 30.24 | 1792 | C_18_ene | 0.49 ± 0.27 | 0.72 ± 0.20 | 1.32 ± 0.41 |  |
| 22 | 34.21 | 2007 | C_20_ene | 0.14 ± 0.19 | 0.14 ± 0.19 | 0.63 ± 0.37 |  |
| 23 | 36.25 | 2100 | *n*-C_21_ | 7.31 ± 3.51 | 4.57 ± 1.31 | 2.02 ± 1.41 |  |
| 24 | 38.01 | 2200 | *n*-C_22_ | 1.41 ± 0.40 | 0.77 ± 0.44 | 0.33 ± 0.61 |  |
| 25 | 39.71 | 2300 | *n*-C_23_ | 23.96 ± 8.57 | 17.82 ± 4.36 | 11.68 ± 2.83 |  |
| 26 | 40.91 | 2369 | 3-MeC_23_ | 3.65 ± 1.70 | 2.23 ± 0.83 | 1.75 ± 0.82 |  |
| 27 | 41.34 | 2400 | *n*-C_24_ | 0.75 ± 0.54 | 0.74 ± 0.39 | 0.61 ± 0.33 |  |
| 28 | 41.51 | 2406 | diMeC_23_ | 2.05 ± 1.42 | 1.91 ± 1.03 | 1.07 ± 0.62 |  |
| 29 | 41.89 | 2430 | dimethyl benzylphenol | 0.12 ± 0.24 | 0.47 ± 0.60 | 0.63 ± 0.75 |  |
| 30 | 42.27 | 2457 | 4-MeC_24_ | 1.17 ± 0.55 | 0.70 ± 0.39 | 0.56 ± 0.28 |  |
| 31 | 42.34 | 2469 | 6,9-C_25_diene | 2.62 ± 1.74 | 3.10 ± 0.77 | 2.50 ± 0.84 |  |
| 32 | 42.44 | 2481 | 9-C_25_ene | 14.07 ± 7.75 | 20.31 ± 3.59 | 17.16 ± 3.54 |  |
| 33 | 42.56 | 2490 | 7-C_25_ene | 1.88 ± 1.07 | 2.88 ± 0.64 | 3.48 ± 1.06 |  |
| 34 | 42.91 | 2500 | *n*-C_25_ | 5.80 ± 1.94 | 7.57 ± 1.82 | 6.92 ± 3.23 |  |
| 35 | 44.03 | 2571 | 3-MeC_25_ | 7.25 ± 2.35 | 5.42 ± 1.72 | 5.96 ± 2.21 |  |
| 36 | 44.56 | 2605 | 3,7/3,9-diMeC_27_ | 4.34 ± 4.53 | 5.92 ± 1.34 | 4.97 ± 1.45 |  |
| 37 | 45.37 | 2671 | 6,9-C_27_diene | 1.95 ± 1.23 | 0.42 ± 0.36 | 1.04 ± 0.43 |  |
| 38 | 45.46 | 2675 | 9-C_27_ene | 6.40 ± 3.82 | 3.19 ± 1.04 | 5.15 ± 2.24 |  |
| 39 | 45.88 | 2700 | *n*-C_27_ | 0.30 ± 0.35 | 0.35 ± 1.01 | 0.32 ± 0.32 |  |
| 40 | 46.22 | 2734 | unknown amide | 0.66 ± 1.09 | 2.11 ± 1.59 | 3.58 ± 1.87 |  |
| 41 | 46.93 | 2773 | 3-MeC_27_ | 0.67 ± 0.53 | 0.38 ± 0.65 | 0.28 ± 0.48 |  |
| 42 | 47.41 | 2805 | diMeC_27_ | 3.09 ± 1.28 | 1.68 ± 0.54 | 1.60 ± 0.88 |  |

| **Table S2**: Mean and standard deviations of the relative contribution (%) of specific volatiles found in the passive headspace of *N. vespilloides* larvae from three different larval instars separated by GC (mean relative peak area). | | | |
| --- | --- | --- | --- |
| Substances | 1st instar | 2nd instar | 3rd instar |
| phenol | 25.20 ± 22.51 | 41.14 ± 21.74 | 65.50 ± 23.63 |
| 2-ethyl-1-hexanol | 1.38 ± 2.59 | 1.02 ± 1.21 | 4.79 ± 13.95 |
| acetophenone | 2.67 ± 2.56 | 4.85 ± 3.19 | 0.87 ± 1.51 |
| phenethyl alcohol | 9.13 ± 10.39 | 1.45 ± 1.04 | 0.45 ± 0.66 |
| ethyl caprylate | 1.58 ± 2.04 | 2.11 ± 1.04 | 3.71 ± 6.69 |
| octanoic acid isopropyl ester | 5.37 ± 4.79 | 4.81 ± 4.07 | 0.63 ± 0.61 |
| quinoline | 0.48 ± 0.79 | 0.91 ± 0.79 | 0.65 ± 0.75 |
| indole | 36.10 ± 23.86 | 20.22 ± 13.65 | 9.18 ± 7.75 |
| methyl geranate | 13.99 ± 16.54 | 23.35 ± 12.11 | 0.68 ± 1.46 |
| hexadecanoic acid | 4.10 ± 15.47 | 0.13 ± 0.52 | 13.53 ± 20.75 |

**Mass spectra of unidentified substances form Table S1**

**
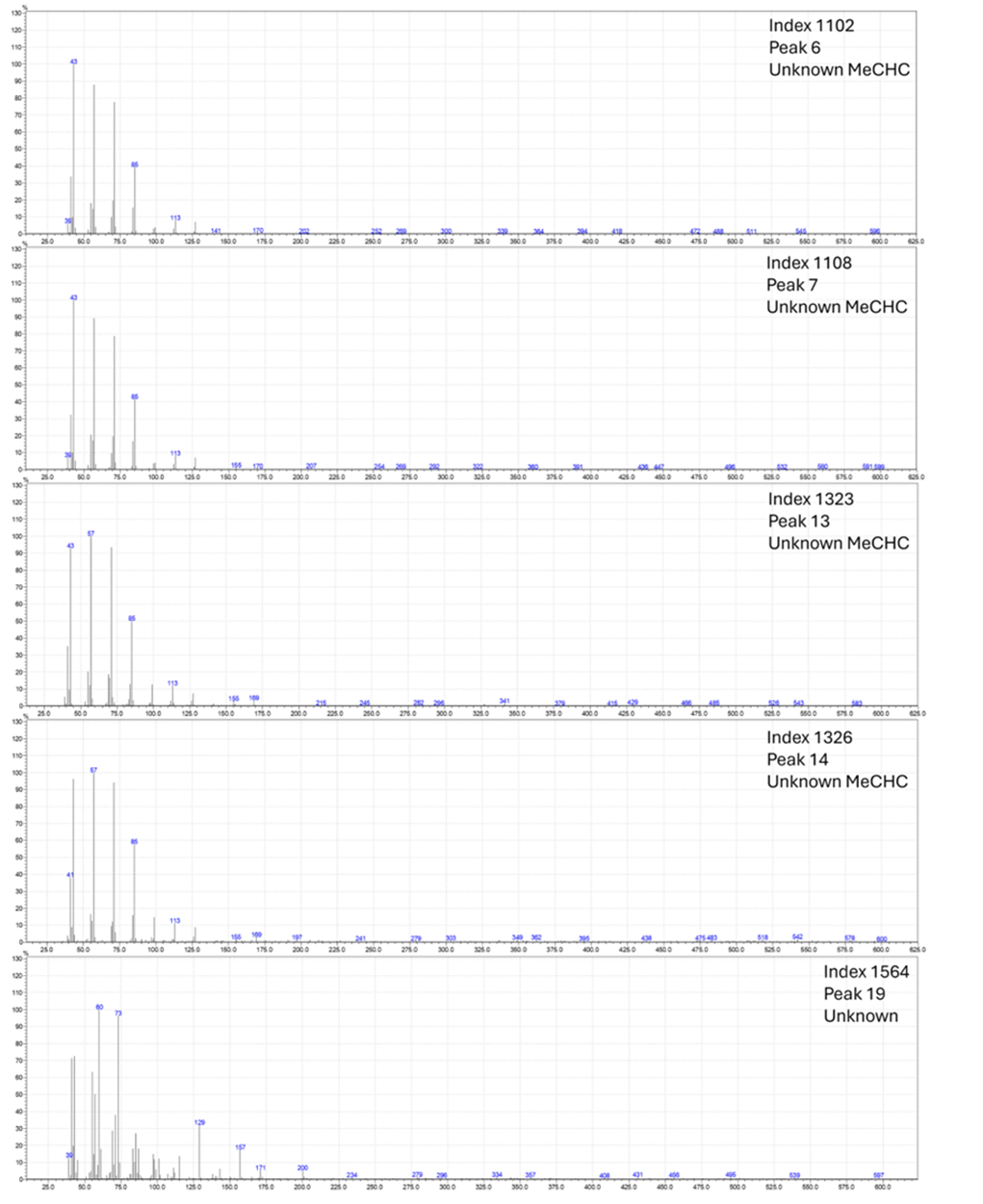
**
